# Human Mammary Tumor Virus (HMTV) sequences in human milk

**DOI:** 10.1186/1750-9378-9-20

**Published:** 2014-06-17

**Authors:** Teiko Nartey, Heberth Moran, Tania Marin, Kathleen F Arcaro, Douglas L Anderton, Polly Etkind, James F Holland, Stella M Melana, Beatriz G-T Pogo

**Affiliations:** 1 The Tisch Cancer Institute and Department of Medicine, Icahn School of Medicine at Mount Sinai, 1 Gustave L. Levy Place, New York, NY 10029, USA; 2 Department of Veterinary and Animal Sciences, University of Massachusetts at Amherst, 240 Thatcher Road, Amherst, MA 01003, USA; 3 Department of Sociology, Sloan College, University of South Carolina, Rm 321. 911 Pickens St., Columbia, SC 29208, USA; 4 Department of Microbiology, Icahn School of Medicine at Mount Sinai, 1 Gustave L. Levy Place, New York, NY 10029, USA

**Keywords:** Retrovirus, Breast cancer, HMTV, Milk, Breast biopsies

## Abstract

**Background:**

Retroviral sequences 90-95% homologous to the mouse mammary tumor virus (MMTV) were present in 38% of the breast cancers studied from American women and were not detectable in non-tumor breast tissue from the same patient. The entire proviral structure was described and viral particles were isolated from primary cultures of human breast cancer. This virus was designated as human mammary tumor virus (HMTV). Hormone response elements present in the HMTV Long-Terminal-Repeat (LTR) suggest a mechanism for association of HMTV with hormonally responding tissues. In fact, the incidence of HMTV sequences is higher in gestational breast cancers, which are associated with hormonal changes. Milk epithelial cells are also under hormonal regulation and therefore are excellent specimens for HMTV sequence detection.

**Methods:**

The HMTV sequence was studied in milk samples from lactating women recruited with increased risk of breast cancer because they had undergone breast biopsies (Biopsy-Group) and lactating women without breast biopsies (Reference-Group).

**Results:**

HMTV-*env* sequences were detected by PCR in milk of 7.61% of 92 women of the Reference-Group and in 20.55% of 73 women of the Biopsy-Group (p: 0.015). The sequences were 94-98% homologous to MMTV. HMTV-*env* and HMTV-*env*/LTR junction sequences were detected in high-speed pellet RNA, implying the presence of HMTV viral particles. PCR assays to detect the murine mitochondrial cytochrome oxidase gene and intracisternal-A-type particle sequences were performed to rule out mouse mitochondrial or genomic DNA contamination. Eight women of the 73 Biopsy-Group participants had breast cancer and the milk of only one of these eight women had HMTV-*env* sequences. In the remaining 65 women of the Biopsy-Group, under enough clinical suspicion to lead to biopsy, HMTV was detected in 14, nearly three times the number of milks as compared to the Reference-Group (21.54% versus 7.61%; p: 0.016).

**Conclusion:**

The significance of HMTV in milk from the Reference-Group, the greater frequency in the milk of women who had undergone a breast biopsy and its possible infectivity for infants are important questions under study. The similarity of HMTV to MMTV is striking and suggests one possible avenue for viral transmission in humans.

## Background

The presence of viral particles with the morphological characteristics of betaretroviruses was first described in human milk by Feller and Chopra [1,2]. In 1971 Moore *et al.*[3] also described a betaretrovirus similar to mouse mammary tumor virus (MMTV) in the milk from 5% of American women with no familial history of breast cancer, 60% of American women with familial breast cancer and 39% from Parsi women [3], who have a three-fold greater risk of developing breast cancer than the non-Parsi population in Bombay [3]. Schlom *et al.* (1972) [4] found Reverse Transcriptase (RT) activity and RNA in viral particles of the same density as that of betaretroviruses in human milk. A human protein related to the envelope protein (Env) of MMTV was identified from a pool of human milk samples from 300 healthy women [5]. Furthermore, another retroviral protein, p27, related to MMTV *gag* gene was also shown to be present in some established breast cancer cell lines and in cells from human milk [6,7]. When human endogenous retroviruses (HERV) with homology to MMTV were described in the 1980’s, it was assumed that the particles described above were endogenous in origin [8]. However, the presence of several other viruses in milk has now been studied [9,10] and a recent report indicated that MMTV-like sequences were present in DNA of milk cells of 5% of healthy Australian Women [11].

We have reported previously the presence of sequences from a retrovirus 90-95% homologous to MMTV in 38-40% of the breast cancers studied in American women [12], whereas these sequences are not detectable in non-tumor mammary tissue from the same breast that contained the tumor [13]. The whole proviral structure has been described and designated as human mammary tumor virus (HMTV) [14]. The presence in human breast cancer of sequences homologous to MMTV has been confirmed by several groups [15-19]. Other authors have designated these sequences as MMTV-like, and thus appear to be the same virus as HMTV [15-19].

HMTV retroviral particles have been isolated from primary cultures of human metastatic breast cancer cells [20] and expression of HMTV proteins has been shown in cells containing HMTV proviral DNA sequences [21]. We have also found that the incidence of HMTV sequences is higher in inflammatory [22] and gestational breast cancers than in sporadic specimens [23]. Gestational breast cancers, by definition, are associated with major hormonal changes and hormone responsive elements present in the LTR of HMTV [23], together suggest a molecular mechanism to explain viral association with hormonally responding tissues. Epithelial cells from milk, because of their association with hormonal changes, are excellent specimens in which to search for HMTV sequences.

In this communication we show that HMTV-*env* sequences are present in the DNA of milk cells from 20.55% of the women who had previously undergone breast biopsies as compared to only 7.61% of the women in the Reference-Group. In addition, the detection of HMTV-*env*/LTR junction sequences from the high-speed pellet RNA indicates the presence of HMTV viral particles in several milk samples of Biopsy and Reference-Group women.

## Results and discussion

Milk samples from 97 women in the Reference-Group and 79 women in the Biopsy-Group were analyzed. The β-globin gene could be amplified in milk samples from 92 of the 97 women in the Reference-Group and 74 of the 79 women in the Biopsy-Group and thus they were qualified for further analysis. All the DNA samples showing presence of viral sequences were tested for the presence of murine mitochondrial cytochrome oxidase gene (MoMt) and murine intracisternal A-particles (IAP) by PCR. Only one sample in the Biopsy-Group was found to contain murine DNA and was eliminated from future study.

**Figure 1:**
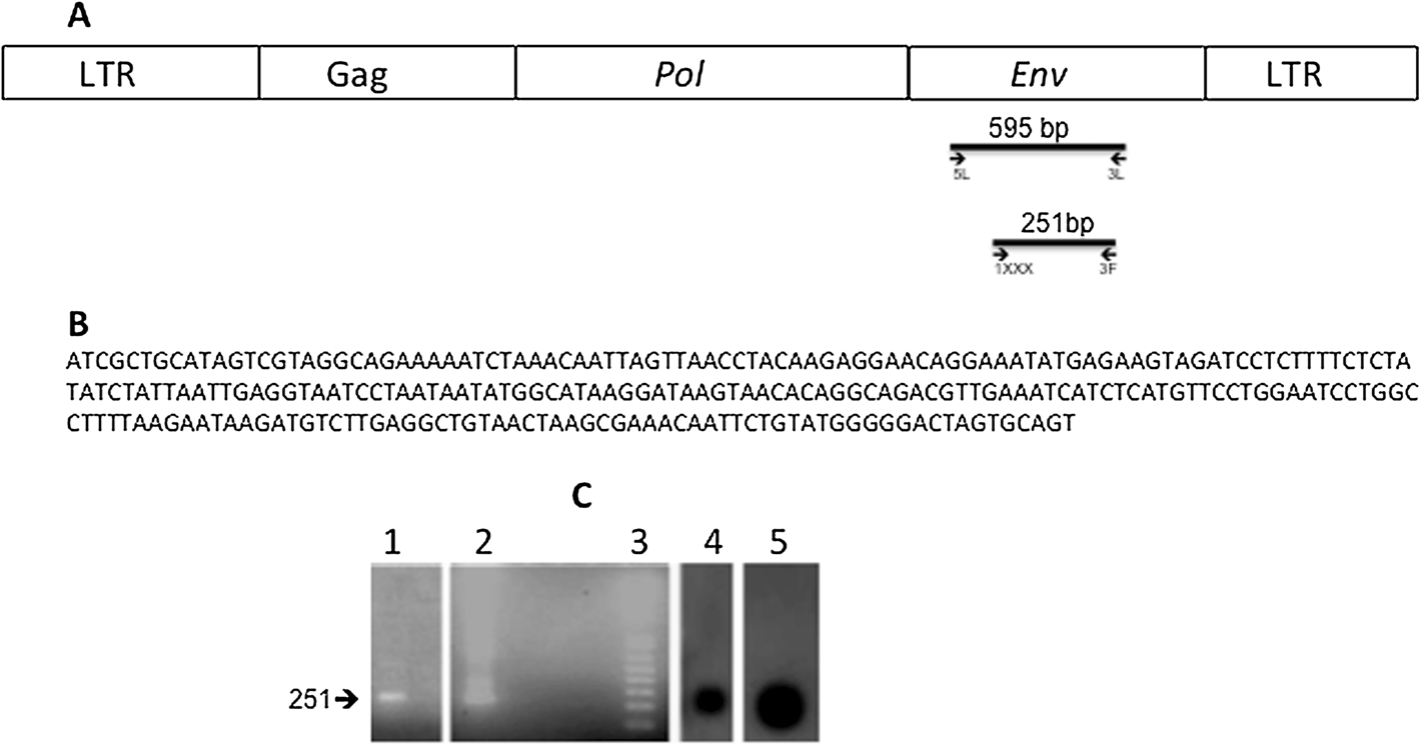
**Detection of HMTV-*env *gene sequences in milk cells.** PCR was performed using primers 5 L and 3 L and nested PCR using primers 1XXX and 3 F as described in Methods. **A**. Diagram of HMTV proviral DNA structure and location of the primers. **B**. HMTV-*env* DNA sequence using primers 1XXX and 3 F in a nested PCR reaction (GenBank KJ831809). **C**. 2% agarose gel electrophoresis of HMTV-*env* 251 bp amplicon. Lane 1: HMTV-*env* positive sample, Lane 2: HMTV-*env* positive control, Lane 3: 1 kb plus DNA ladder. Southern blot hybridization using ^32^P labeled probe as described in Methods. Lane 4: HMTV-*env* positive sample. Lane 5: HMTV-*env* positive DNA control.

The results (Table 1) indicated that HMTV-*env* was detected in the DNA of 7 of 92 (7.61%) Reference-Group women. For the Biopsy-Group, 15 of the 73 women (20.55%) had HMTV sequences in the DNA of their milk cells. This difference between both groups was statistically significant (p: 0.015). The higher percentage of women with HMTV sequences in their breast milk DNA was not correlated with any of the demographic parameters previously examined including age at time of milk donation, time after delivery, number of live births, or age at first pregnancy [24,25]. Eight women in the Biopsy-Group were diagnosed with breast cancer and one of them was found to have HMTV sequences in her milk cell DNA. Fourteen of the 65 (21.54%) biopsied women without breast cancer had HMTV-*env* sequences in their milk. One representative experiment is shown in Figure 1.

**Table 1:** **Presence of HMTV-*env *** **sequences in human milk genomic DNA (P1)**

**Group**	**Number of women**	***env *** **sequences**	**%**	P*
Reference	92	7	7.61	-
Biopsied	73	15	20.55	0.015

*The chi-square test was used to compare the two groups.

When HMTV-*env* amplicon sequences were compared to MMTV (GenBank: M15122) sequences reported in the GenBank they were found to be 94 to 98% homologous. No major differences were found between HMTV sequences isolated from the women in either group. The HMTV-*env* sequence was submitted to the GenBank.

**Figure 2:**
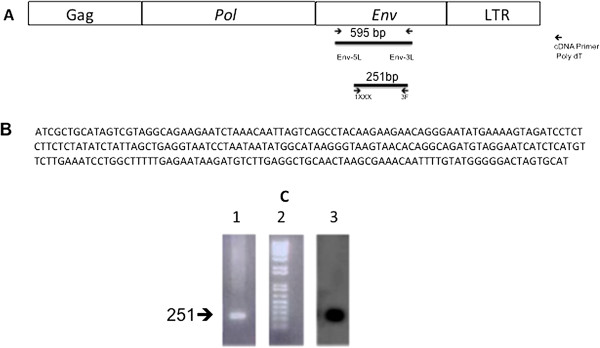
**Detection of HMTV-*env *gene sequences in P2 RNA.** cDNA synthesized from high speed pellet (P2) RNA using polydT as primer, PCR was performed using primers 5 L and 3 L and nested PCR using primers 1XXX and 3 F as described in Methods. **A**. Diagram of HMTV viral RNA structure and location of the primers. **B**. HMTV-*env* cDNA sequence was amplified using primers 1XXX and 3 F in a nested PCR reaction (GenBank KJ831810). **C**. 2% agarose gel electrophoresis of HMTV-*env* 251 bp amplicon. Lane 1: HMTV-*env* positive sample. Lane 2: 1 kb plus DNA ladder. Lane 3: Southern blot hybridization using ^32^P end labeled probe for HMTV-*env* positive sample.

**Figure 3:**
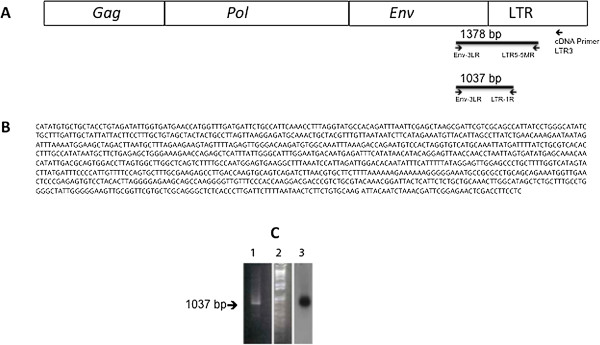
**Detection of HMTV- *env */LTR junction sequences in P2 RNA.** cDNA synthesized from high speed pellet (P2) RNA using primer LTR3 and PCR performed using primers ENV-3LR’, LTR-5MR and semi nested PCR with ENV-3LR’ and LTR-1R’ as described in Methods. **A**. Diagram of HMTV viral RNA structure and location of the primers. **B**. Sequence amplified from cDNA using primers ENV-3LR’, LTR-5MR by PCR and by semi nested PCR with ENV-3LR’ and LTR-1R’ primers (GenBank KJ831811). **C**. 1% agarose gel electrophoresis of HMTV-*env*/LTR 1018 bp amplicon. Lane 1: HMTV-*env*/LTR positive cDNA sample_._ Lane 2: 1 kb plus DNA ladder. Lane 3: Southern blot hybridization using ^32^P end labeled probe for HMTV*-env*/LTR positive cDNA sample.

HMTV particle sequences were also sought in the high-speed pellet (P2) RNA from milk of the same Biopsy and Reference Group women whose milk cell DNA had HMTV sequences. RNA was extracted as described in Methods and RT-PCR carried out for HMTV-*env* (Figure 2) and HMTV-*env*/LTR junction sequences (Figure 3). The HMTV sequences from the cDNA were compared to sequences present in the GenBank. HMTV-*env* sequences were 94 to 98% homologous to MMTV (GenBank: M15122) and HMTV-*env*/LTR junction sequence was 96% homologous to MMTV (GenBank: M15122). Results in Table 2 indicate that HMTV-*env* and HMTV-*env*/LTR junction sequences were amplified from P2 RNA when the HMTV-*env* also was detected in the genomic DNAs from P1. HMTV-*env* sequences were amplified in P2 RNA from 2 out of 3 women of the Reference-Group. All three of these women also had HMTV sequences detected in their genomic DNA and HMTV-*env*/LTR junction sequences present in P2 RNA. In the case of the Biopsied-Group, HMTV-*env* and HMTV *env*/LTR junction sequences were amplified in P2 RNA from 4 out of 5 women whose genomic DNA contained HMTV sequences. HMTV-*env*/LTR junction sequences were detected in a total of 7 P2 RNA samples of which one contained enough material to allow sequencing. HMTV-*env* and *env*/LTR junction sequences from P2 RNA have been submitted to the GenBank. Absence of glycerol-3-phosphate dehydrogenase gene (G3DPH) mRNA from high-speed pellets and the presence of HMTV-*env*/LTR junction sequences is consistent with the presence of virion RNA.

**Table 2:** Detection of HMTV sequences in human milk high speed Pellet P2 RNA

**Group**	**HMTV-*env *** **sequences in genomic DNA (P1)**	**HMTV-*env *** **sequences in RNA (P2)**	**HMTV- *env */LTR junction sequences in RNA (P2)**
Reference	3	2/3	3/3
Biopsied	5	4/5	4/5

During lactation, the mammary gland undergoes molecular changes in response to hormonal influences. Gestational breast cancer that develops during pregnancy or lactation is considered to be very aggressive and related to hormonal changes, and it has been shown to have a high incidence of viral HMTV sequences [23].

In this communication evidence is presented that milk cells of 7.61% of women from a Reference-Group contain HMTV viral sequences in their genomic DNA. The percentage of women with HMTV sequences in the milk cells from the Biopsy-Group is remarkably higher (20.55%). The usual indications for biopsy are physical, radiologic, or sonographic findings, which raise the possibility of breast cancer [26]. According to the American Cancer Society, roughly one out of five breast biopsies results in a diagnosis of cancer [26]. Eight of the women in the Biopsy-Group were found to have breast cancer and one of the eight had HMTV sequences in her milk. Of the remaining 65 women of the Biopsy-Group, under enough clinical suspicion to lead to biopsy, 21.54% contain HMTV sequences in their milks. This percentage represents a nearly three-fold increase compared to the Reference-Group women (p: 0.016). This finding suggests that the presence of the HMTV sequences is associated with increased breast cancer risk.

The present results confirm, extend, and validate the findings of the 1960s and 1970s that provided morphological and immunologic evidence of viral particles similar to MMTV in human milk [1-4]. These results are in agreement with the observations of Johal *et al.*[11] that *env* sequences are present in the milks from 5% of Australian healthy women. We have proven that the sequences are not derived from murine contamination, and add the striking observation that clinical findings that led to a biopsy were associated with a nearly 3-fold increase in women who had HMTV sequences in their milk. Furthermore, we have also shown that there is HMTV-*env*/LTR junction sequences in the high-speed pellet RNA from milk supernatants, which is consistent with the presence of viral particles.

## Conclusion

The significance of HMTV in human milk, its greater frequency in the milk of women who have undergone a breast biopsy and its possible transmission to their infants, are important questions still under study. The similarity of HMTV and MMTV in this regard is striking and suggests one possible avenue for human transmission.

Breastfeeding in general has been considered to lower the risk of eventual breast cancer in the offspring [27]. However, nearly all infants have been exposed to colostrum, even when mothers deny having breastfed their children [11,28,29]. Therefore, a small proportion of breastfed infants who might have been at increased risk may have been hidden in the data. The results presented here pave the way to stimulate research in this area and to develop strategies that are precisely targeted to prevent transmission by HMTV carrying women, without eliminating the well-known benefits of breastfeeding for those who do not carry the virus. Such attempts have been very successful in Japan, where HTLV, the causative agent of adult T-cell leukemia, has been significantly reduced in the population by measures that dissuade HTLV carrier mothers from breastfeeding [30].

## Methods

### Milk sample collection

Milk samples were provided by K. F. Arcaro at the University of Massachusetts at Amherst, MA. Collection was performed with the women’s consent, following the conditions approved by the institutional review board (IRB) of the University of Massachusetts-Amherst as described in previous publications [24,25]. Milk from two groups of American women was studied. One group, referred to as the Reference-Group, involved nursing women without biopsies. The other group, referred to as the Biopsy-Group, consisted of nursing women from across the US who either had undergone a breast biopsy at some point in their lives or were scheduled to have a breast biopsy. The Biopsy-Group represents women whose clinical or radiological findings suggested the need for a biopsy to exclude breast cancer and are thus considered to be at increased risk of breast cancer [25]. Frozen milks were sent on dry ice to B. G-T Pogo. For each of 97 women in the Reference-Group, a single sample (80 to 120 ml) of milk was received. For 60 women in the Biopsy-Group matched samples (80 to 120 ml) of milk from the left and right breasts were received along with a single sample of similar volume from either the left or right breasts of an additional 19 women. Thus a total of 139 samples from 79 women was received from the Biopsy-Group. Milks were kept frozen at -20°C until processing. The milk samples were first centrifuged at 1,000 × g for 30 minutes at 4°C. The fat layer was removed and supernatant designated S1 transferred to a new tube and the resulting cell pellet (P1) was re-suspended in PBS and DNA was extracted. The supernatant was then centrifuged at 10,000 × g for 30 min. and the resulting supernatant was centrifuged at 100,000 × g for 150 min at 4°C. This high-speed pellet designated as P2 was stored at -80°C.

### DNA extraction

DNA from P1 was extracted according to the manufacturer’s instructions (Life Technology Inc., Carlsbad, CA). The suitability of DNAs for PCR was tested by amplifying a 250 bp sequence of the β-globin gene using primers GH-20 (5′GAAGAGCCAAGGACAGGTAC-3′) and PCO4 (5′CAACTTCATCCACGTTCACC-3′) under the conditions previously described [31]. β-globin sequences were amplified in all reported specimens.

### Detection of HMTV-*env* sequences

PCRs were performed on 250 ng of DNA using Illustra PureTaq Ready-To-Go PCR beads (GE Healthcare Inc., Piscataway, NJ) and 10 pmol of the following primers 5 L (5′-CCAGATCGCCTTTAAGAAGG-3′) and 3 L (5′-TACAGGTAGCAGCACGTATG-3′). The amplicon product was 595 bp. A nested PCR was then performed with the primers 1XXX (5′-ACTGCACTAGTCCCCCATAC-3′) and 3 F′ (5′-ATCGCTGCATAGTCGTAGGC-3′). The product is 251 bp. Both HMTV-*env* PCR reactions were performed in a thermocycler under the following conditions [32] with minimal modifications: a cycle of 95°C for 5 min, followed by 35 cycles of 95°C for 30 sec, 58°C for 30 sec, 72°C for 30 sec, and a final step of 72°C for 7 min. Each sample was tested three times to assure the reproducibility of the reaction. In addition, several reactions without template DNA were performed, along with the experimental reactions, to rule out possible contamination of the nuclease-free sterile water and primers.

To identify the amplicon, the gel was transferred onto a positively charged nylon membrane (GE Healthcare Inc., Piscataway, NJ) by capillary action and hybridized with ^32^P-labeled oligonucleotide 1XXXX probe (5′-AGGCCAGGATTTCAAGAACA-3′). The probe was radiolabeled by the end terminal labeling method (New England Biolabs, Ipswich, MA).

After the band was identified by hybridization the remaining PCR product was amplified using the same nested primers and again run in an agarose gel. The nested PCR product (251 bp) was removed from the gel, purified following the conditions recommended by the manufacturer of QIAQuick Gel Extraction Kit (Qiagen Inc., Valencia, CA), and submitted for sequencing to the Mount Sinai Genomics Institute Sequencing core facility.

### RNA extraction

RNA was extracted from the 100,000 × g pellet (P2) using Trizol LS Reagent following the manufacturer′s protocol (Life Technologies, Carlsbad, CA). RNA samples were treated with DNAse and the absence of DNA was confirmed by failing to detect the G3DPH gene sequence by PCR using GE Healthcare Illustra PureTaq Ready To Go PCR Beads, (GE Healthcare, Piscataway NJ), 10 pmol of primers: G3DPH-3 (5′-GGTGAAGACGCCAGTGGACTC-3′), and G3DPH-5 (5′-GTGAAGGTCGGAGTCAACGGA-3′) and the conditions previously described [20].

### cDNA preparation

Two sets of cDNA were synthetized using the same P2 RNA samples. One set of cDNA synthesized with oligo-dt was used for the detection of G3DPH and HMTV-*env* and the other set with LTR3 primer was used for the detection of HMTV-*env*/LTR junction sequences.

#### Oligo-dt cDNA synthesis

Oligo-dt cDNA was obtained using 2 ug of RNA, oligo-dt primer (Thermo Fisher Scientific, Pittsburgh, PA), and RNaseOut (Life Technologies, Grand Island, NY) following the protocol of the Omniscript Reverse Transcription Kit (Qiagen Inc., Valencia, CA) [20].

### Detection of HMTV-env/LTR junction sequences

cDNA from HMTV-LTR (LTR3 cDNA) was obtained using 2 µg of P2 RNA, LTR3 primer (5′-CGAACAGACACAAACACACG-3′), and RNaseOut following the protocol of the Qiagen Omniscript Reverse Transcription Kit [20].

HMTV-*env*/LTR junction sequences were detected in the LTR3 cDNA by PCR using 10 pmol of primers LTR-5MR (5′-ATAAGTCCCTGGTTGCCACC-3′) and ENV-3LR′ (5′-CATATGTGCTGCTACCTGTA-3′), 250 ng of cDNA and the Advantage 2 PCR Kit (Clontech Laboratories, Inc., Mountain View, CA) under the following conditions: 95°C for 1 min, 35 cycles of 95°C for 30 sec, 62°C for 1 min, 68°C for 1 min, and finally a cycle of 68°C for 10 min. The expected amplicon was 1378 bp. A semi-nested PCR was then performed with the primers ENV-3LR′ and LTR-1R′ (5′-TCAGGAGGAAGGTCGAGTTCT-3′) under the following conditions: 95°C for 1 min, 35 cycles of 95°C for 30 sec, 62°C for 1 min, 68°C for 1 min, and finally a cycle of 68°C for 10 min [20]. The product was 1018 bp. Each sample was tested three times to assure the reproducibility of the reaction. In addition, several reactions without template DNA were performed, along with the experimental reactions, to rule out possible contamination of the nuclease-free sterile water and primers. To identify the amplicon, the gel was then transferred and hybridized with a ^32^P-labeled oligonucleotide env/LTR probe (5′-CTGCAGCAGAAATGGTTGAA-3′) [20].

After amplicons were detected on film, the remaining PCR products were re-amplified using the same primers. The amplified PCR product was run in a 1% agarose gel, isolated and purified using the Quick Gel Extraction Kit and submitted for sequencing at the Mount Sinai Genomics Institute Sequencing facility. The sequences were compared to sequences present in the GenBank. All procedures were carried out in a laminar flow hood based in a laboratory with exclusive use for human tissues.

### Detection of murine DNA

Although the experiments were performed in a room restricted to human tissue work, a series of PCRs were done to detect mouse DNA contamination in any human DNA in which HMTV sequences were detected. Two assays were used to detect murine contaminant DNA [32].

One of these assays is for the detection of cytochrome oxidase, a gene that is part of MoMt [32]. To test for MoMt, the following primers were used: mt15982F (5′- AGACGCACCTACGGTGAAGA-3′) and mt16267R (5′-AGAGTTTTGGTTCACGGAACATGA-3′) [32]. The product yields an amplicon of 286 bp. A semi-nested PCR was then performed using the primers mt16115F (5′-TGCCAAACCCCAAAAACACT-3′) and mt16267R, which results in a 153 bp amplicon [32]. The PCR product was transferred from the gel to a positively charged nylon membrane and hybridized with a ^32^P-labeled oligonucleotide MoMt DNA probe (5′-GAACTAGAATTGATCAGGCAT-3′). The sensitivity of this reaction has been improved by the hybridization step and is 1.25 fg.

The other assay is for the detection of IAP. IAP are retrotransposon elements present at the level of about 1000 copies of varying length per mouse genome. IAP PCR has been proposed as an assay for the detection of murine DNA contamination of human DNA specimens [33]. Amplification of the IAP sequences from the mouse genome by PCR was carried out using the following primers: forward primer (5′-ATAATCTGCGCATGAGCCAAGG-3′) and reverse primer (5′-AGGAAGAACACCACAGACCAGA-3) under the recommended conditions [33]: one cycle of 95°C, 5 minutes, 35 cycles of 95°C 30 seconds, 58°C 30 seconds, 72°C 20 seconds and one cycle of 72°C, 7 minutes. Products of variable size, reflecting diversity of the IAP, were visualized in a 2% ethidium bromide stained agarose gel [33]. The sensitivity of this reaction is 1.25 pg.

### Statistical analysis

Statistical analysis was performed using a Chi-square test to determine the association of the presence of HMTV sequences in the DNA of milk cells from the women of the Biopsy or Reference Group [34]. This test does not introduce multivariate control for other possible differences between Biopsy and Reference Groups. However, other covariates were also examined in bivariate tests (which adjusted for sample clustering differences between the two groups of subjects when necessary) and were not found to be significantly associated with the presence of HMTV sequences.

## Abbreviations

MMTV: Mouse mammary tumor virus; HMTV: Human mammary tumor virus; LTR: Long-Terminal-Repeat; Env: Envelope; PCR: Polymerase chain reaction; RT: Reverse transcriptase; HERV: Human endogenous retrovirus; MoMt: Murine mitochondrial DNA; IAP: Intracisternal-A-type particles; G3PDH: Glycerol-3-phosphate dehydrogenase; HTLV: Human T cell leukemia virus; IRB: Internal review board.

## Competing interests

The authors declare no competing interest.

## Authors’ contributions

TN, HM and TM performed the laboratory experiments; KFA was responsible for supplying the milk specimens. DLA carried out the statistical analysis; SMM and BGTP designed the experiments and supervised the experiments. SMM, BGTP, PE and JFH were involved with the writing of manuscript. All authors read and approved the manuscript.
